# Major zoonotic diseases of public health importance in Bangladesh

**DOI:** 10.1002/vms3.465

**Published:** 2021-03-02

**Authors:** Sukanta Chowdhury, Mohammad A. Aleem, Md Shafiqul I. Khan, Mohammad Enayet Hossain, Sumon Ghosh, Mohammed Z. Rahman

**Affiliations:** ^1^ International Centre for Diarrhoeal Disease Research, Bangladesh (icddr,b) Dhaka Bangladesh; ^2^ University of New South Wales (UNSW) Sydney NSW Australia; ^3^ Department of Food Microbiology Patuakhali Science and Technology University Patuakhali Bangladesh

**Keywords:** Bangladesh, one‐health approach, public health, zoonotic diseases

## Abstract

Zoonotic diseases cause repeated outbreaks in humans globally. The majority of emerging infections in humans are zoonotic. COVID‐19 is an ideal example of a recently identified emerging zoonotic disease, causing a global pandemic. Anthropogenic factors such as modernisation of agriculture and livestock farming, wildlife hunting, the destruction of wild animal habitats, mixing wild and domestic animals, wildlife trading, changing food habits and urbanisation could drive the emergence of zoonotic diseases in humans. Since 2001, Bangladesh has been reporting many emerging zoonotic disease outbreaks such as nipah, highly pathogenic avian influenza, pandemic H1N1, and COVID‐19. There are many other potential zoonotic pathogens such as Ebola, Middle East respiratory syndrome coronavirus, Kyasanur forest disease virus and Crimean–Congo haemorrhagic fever that may emerge in the future. However, we have a limited understanding of zoonotic diseases’ overall risk in humans and associated factors that drive the emergence of zoonotic pathogens. This narrative review summarised the major emerging, re‐emerging, neglected and other potential zoonotic diseases in Bangladesh and their associated risk factors. Nipah virus and *Bacillus anthracis* caused repeated outbreaks in humans. More than 300 human cases with Nipah virus infection were reported since the first outbreak in 2001. The highly pathogenic avian influenza virus (H5N1) caused more than 550 outbreaks in poultry, and eight human cases were reported so far since 2007. People of Bangladesh are frequently exposed to zoonotic pathogens due to close interaction with domestic and peri‐domestic animals. The rapidly changing intensified animal–human–ecosystem interfaces and risky practices increase the risk of zoonotic disease transmission. The narrative review's findings are useful to draw attention to the risk and emergence of zoonotic diseases to public health policymakers in Bangladesh and the application of one‐health approach to address this public health threat.

## Graphical abstract

Live poultry market ‐ an important interface for zoonotic pathogens spillover.

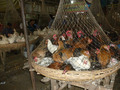

## 1 INTRODUCTION

Zoonotic diseases develop mild‐to‐severe illnesses in humans transmitted from vertebrate animals (Slingenbergh et al., 2004). The majority of the human diseases originate from animals (61%), and 70% of them are emerging diseases (Jones et al., 2008; Slingenbergh et al., 2004; Wang & Crameri, 2014). Most of the emerging zoonotic diseases, including highly pathogenic avian influenza, nipah virus, MERS‐CoV, SARS, COVID‐19 and pH1N1 (pandemic H1N1) caused severe infections in humans globally. Domestic and wild animals act as a reservoir for zoonotic diseases. Domestic animals such as livestock, pet and poultry transmit pathogens frequently to humans because of close interaction (Han et al., 2016). Rodents are very abundant, peri‐domestic in nature and contributed more than 80 zoonotic pathogens to humans (Han et al., 2016). Bats were identified as a reservoir host for many emerging diseases in humans such as Nipah, MERS‐CoV, SARS, Rabies and Ebola (Wang & Crameri, 2014). The zoonotic disease can be categorised into emerging, reemerging and neglected. Many zoonotic diseases were detected worldwide, and some were regional. Most zoonotic animal host species such as domestic livestock, pets, poultry, bats and rodents are abundant in Southeast Asia, Central and South America, Eastern Europe and Central East Africa (Han et al., 2016).

Many anthropogenic factors include changes in human habitat and behaviour, animal–human interaction, urbanisation, modernisation of agriculture and livestock farming, wild animal trade, wildlife hunting, climate change, destruction of wild animal habitat and mixing wild and domestic animals contributed the emergence of zoonotic diseases to human. Along with these anthropogenic factors, intrinsic factors such as hosts, pathogens and vectors also contributed to zoonotic pathogens’ spillover to humans (Han et al., 2016). Trans‐disciplinary efforts can be more useful to detect, prevent and control zoonotic diseases globally. Recently, national, international (the Food and Agriculture Organization of the United Nations, the World Organization for Animal Health, and the World Health Organization) and professional agencies recognised a new multi‐sectoral one‐health approach to address the public health threat of animal origin. This one‐health approach emphasises the interconnectedness of human, animal, environment and eco‐health (Kahn, 2011; Karesh et al., 2012; Rabinowitz et al., 2013). We found many pieces of evidence for the effectiveness of this collaborative approach between human and animal health services to address not only disease threats but also predict certain diseases (Cleaveland et al., 2003; Guan et al., 2007; Kwan et al., 2012; Mazet et al., 2009; Schelling et al., 2007).

Bangladesh is considered as one of the critical global hotspots for zoonotic spillover to humans (Allen et al., 2017). Human behaviour, high abundance of animal and human population, greater extent of the animal–human interface, many live animal markets, wildlife diversity, urbanisation, deforestation and fragile ecosystem may increase the risk of zoonotic disease emergence. The Government of Bangladesh selected six diseases, including anthrax, brucellosis, nipah virus, rabies, zoonotic influenza and zoonotic tuberculosis, as priority zoonotic diseases through a one‐health zoonotic disease prioritisation workshop (CDC, 2017). HPAI viruses caused repeated outbreaks in chicken and more than 550 outbreaks were reported since 2007 (OIE, 2020). More than 300 human cases with Nipah virus were reported since 2001 (IEDCR, 2020). Human anthrax cases were identified every year (IEDCR, 2015). As a low resource country, limited studies and surveillance were conducted to understand zoonotic diseases' burden. The overall risk of zoonotic diseases on public health and disease emergence factors is not well summarised. People of Bangladesh live very close to their domestic livestock and poultry. Slaughtering and selling sick animals are not uncommon. Agriculture farmers, farm workers, butchers and live animal market workers are at high risk of frequent exposure to animals. People are not well aware of the risk of zoonotic disease transmission. A better understanding of the public health impact of zoonotic diseases and associated factors is needed to identify the knowledge gap and assist in developing interventions. This narrative review attempted to explore the past zoonotic events, distribution of zoonotic pathogens, potential risk factors and future risk for the emergence of potential zoonotic diseases. This review paper provided a detailed overview of reported zoonotic diseases of public health significance in Bangladesh that will assist in public health decision‐making.

## 2 METHODS

We conducted a narrative review of the published literature with a focus on zoonotic diseases reported from Bangladesh. Relevant research articles, review articles, abstracts, case reports, communications, letters, book chapters, conference proceedings and other relevant documents were searched in MEDLINE, PubMed, PubMed Central and Google Scholar. Specific keywords such as “zoonotic diseases” or “zoonotic Bangladesh” or “emerging diseases Bangladesh” or “disease name and Bangladesh” or “pathogen name and Bangladesh” were used to search relevant articles. Articles that were published between January 1972 and April 2020 were considered for this review as Bangladesh was turned into an independent sovereign country after the 1971 liberation war. Initially, more than 300 publications were identified and listed to review only abstract or summary by all authors. After initial screening, more than 150 publications were selected for the full review. The first author reviewed thoroughly all selected articles, abstracts and published documents that were informative and useful for this paper. Finally, all extracted data were reviewed and revised based on feedback from all co‐authors.

## 3 RESULTS AND DISCUSSION

In the first stage, we identified more than 330 publications that were related to the Bangladesh context. A total of 163 publications were selected finally to extract data. We found the highest number of publications from Bangladesh on the nipah virus followed by avian influenza. In 2017, the government of Bangladesh highlighted six zoonotic diseases to control on a priority basis. There are some other neglected but potential zoonotic diseases that were reported from Bangladesh. Many potential zoonotic diseases have not been reported yet but may emerge in the future. This review paper summarised all identified and future potential zoonotic diseases that could threaten public health locally and globally.

### 3.1 Highly pathogenic avian influenza

Highly pathogenic avian influenza (HPAI) has caused repeated outbreaks in poultry and sporadic infection in humans globally (OIE, 2020; WHO, 2020a). H5 and H7 subtypes of type A influenza virus under the *Orthomyxoviridae* family are known to be highly pathogenic in poultry (Swayne & Suarez, 2000). Chicken is the most susceptible host with high case fatality (Alexander, 2007). Wild birds, including shorebirds, gulls and domestic ducks act as a reservoir for HPAI. Bangladesh reported the first HPAI (H5N1) outbreak in poultry in March 2007. More than 550 outbreaks were reported in poultry from 2007 to 2019 (OIE, 2020). Commercial chicken farms were mostly affected. Mortality was also observed in backyard chicken flocks, turkeys, ducks, geese and crows (Haider et al., 2017). The first human case of H5N1 was identified in January 2008 (Brooks et al., 2009). As of 30 April 2020, eight human cases with H5N1 infection were reported with one case‐fatality. Eight different clades (2.2, 2.2.2, 2.2.3, 2.3.2, 2.3.4, 2.3.2.1, 2.3.2.1a and 2.3.4.2) of the H5N1 virus were detected from 2007 to 2019 (Chowdhury et al., 2019). The live poultry market is considered a high‐risk area for HPAI virus transmission in poultry and humans (Khan et al., 2018). The environment of poultry shops that slaughtered poultry within their shop was more contaminated with influenza A viruses compared with shops that did not allow slaughter (Chowdhury et al., 2020). Poultry workers at live poultry market are frequently exposed to avian influenza viruses, and antibody against the H5N1 virus was detected in 2% of poultry workers. Poultry workers who fed poultry, cleaned faeces and utensils and handled sick poultry were at high risk of getting H5N1 infection (Nasreen et al., 2015). Commercial chicken farms have been using vaccines against H5N1 since 2012 to reduce mortality. Many epidemiological and molecular studies were conducted to characterise avian influenza viruses and identify risk factors for avian influenza. However, compared with other parts of the world, Bangladesh has experienced a low number of human cases for H5N1 with lower case fatality. Several factors including baseline immune status of people in the country have been postulated behind humans’ low prevalence. The ongoing hospital‐based influenza surveillance might not be sensitive enough to capture the countrywide existence of human cases of avian influenza. Perhaps a seroprevalence study among people residing in hotspots of avian influenza prevalence could identify people with past exposure. Despite vaccination against the H5N1 virus in commercial chicken, HPAI viruses including H5N1 continue to circulate. Small scale poultry farmers and market workers are very reluctant to follow proper biosecurity and biosafety practices. Live poultry markets should introduce centralised slaughter facilities to reduce environmental contamination. Poultry farm biosecurity practices need to be monitored on a regular basis by government livestock officials. More research works are yet to be conducted to reveal human immune responses to avian influenza viruses and evaluate the effectiveness of ongoing avian influenza vaccination. An integrated one‐health surveillance (OHS) is necessary to detect novel influenza viruses of public health importance at the animal–human interface. This OHS platform collects data from multiple domains such as humans, animals (domestic and wildlife), and the environment through cross‐sectoral collaboration.

### 3.2 Nipah virus infection

Nipah virus (NiV) infection is a highly fatal emerging zoonotic disease caused by an emergent Henipavirus under the family *Paramyxoviridae* (Chua et al., 2000). The first outbreak was identified in Malaysia in 1998. More than 265 human cases, including 105 deaths, were reported in Malaysia and Singapore. Pig farmers were mostly infected with NiV in Malaysia (Lam & Chua, 2002). Pig farmers received the infection from NiV‐infected pigs. The role of pigs in Malaysia was intermediate as well as dead‐end host. Fruit bats were identified as the natural reservoir of this virus (Epstein et al., 2016; Lam & Chua, 2002). Bangladesh first reported the NiV outbreak in 2001. Since then, the country has reported outbreaks almost every year with a seasonal pattern (Luby et al., 2009). More than 300 human cases were reported from 2001 to 2020, and the case fatality rate was 70% (IEDCR, 2020). In Bangladesh, most cases of NiV outbreaks were detected during winter (December to March; Luby et al., 2009). Fruits bats of Bangladesh were identified as a natural reservoir for NiV (Anderson et al., 2019; Epstein et al., 2016). Other henipaviruses (Hendra and Cedar viruses) have not been detected so far in bats (Epstein et al., 2020). However, an epidemiological study detected antibodies against henipa‐like viruses in domestic animals of Bangladesh (Chowdhury et al., 2014). Nipah virus infection in humans in Bangladesh mostly associated with drinking NiV contaminated raw date palm sap by fruit bats during winter (Khan et al., 2010; Luby et al., 2006). Person–person transmission also occurred in Bangladesh (Gurley et al., 2007; Nikolay et al., 2019). NiV is continuing to evolve in Bangladesh. Many studies were conducted so far in Bangladesh to understand epidemiology. The role of domestic animals as intermediate or dead‐end hosts is not well known. Information about effective interventions to reduce transmission from bats to humans is very limited. No vaccine is available yet. Some research studies have been initiated recently to develop vaccines against NiV. People should avoid drinking raw date palm sap to prevent NiV transmission. More efforts are needed to conserve bats’ habitats by reforestation in reducing bats’ contact with human and domestic animals. Institute of Epidemiology, Disease Control and Research (IEDCR) in collaboration with International Centre for Diarrhoeal Disease Research, Bangladesh (ICDDR,B) has been conducting hospital‐based surveillance since 2006 to identify NiV encephalitis cases (Sazzad et al., 2015). Countrywide extensive surveillance will help to detect outbreaks rapidly and identify strain diversity of the NiV in humans and animals (wildlife and domestic) using a multi‐sectoral one‐health approach.

### 3.3 Anthrax

Anthrax is primarily a disease of ruminants caused by the spore‐forming, aerobic, gram‐positive, non‐motile bacterium *Bacillus anthracis*. Anthrax is endemic in Bangladesh. Cattle, sheep and goats are mostly affected. In 1980, anthrax was first reported in man and animals in Bangladesh (Samad & Hoque, 1986). The case fatality rate may reach up to 100% in cattle (Schild et al., 2006). Anthrax outbreaks in animals were mostly detected in Sirajganj, Pabna, Bogra and Meherpur districts of Bangladesh (Biswas et al., 2012; Hassan et al., 2015; Siddiqui et al., 2012). Humans can be exposed to anthrax by slaughtering, handling and eating the meat of infected animals. An anthropological investigation found that people in the affected communities had no awareness about anthrax transmission from infected animals to humans (Chakraborty et al., 2012). Slaughtering sick animals and selling meat from sick animals at a lower price were commonly observed in Bangladesh (Islam, Hossain, et al., 2013). In most cases, people do not follow proper carcass disposal and they throw the carcass in the open field, river, canal and flood water that contaminate the grazing field and environment (Chakraborty, 2010). From 2009 to 2015, multiple anthrax outbreaks were reported in humans; >1,500 cases were reported with no death and all cases were the cutaneous type (Chakraborty et al., 2012; IEDCR, 2015; Siddiqui et al., 2012). No human anthrax cases were reported from 1986 to 2008 (Chakraborty et al., 2012). Anthrax is a vaccine‐preventable disease in the animal and the Livestock Research Institute of Bangladesh produces anthrax vaccines for animals (Islam et al., 2017). Human infection can be prevented by vaccinating all susceptible animals. The annual vaccine production was about 4 million doses, which are not adequate to vaccinate all susceptible animals. The government should increase vaccine production to achieve 80%–100% vaccination coverage. The effectiveness of the existing vaccine needs to be evaluated routinely to ensure solid immunity. Awareness about anthrax transmission from a sick animal to humans and the importance of animal vaccination can reduce anthrax infection in humans and animals. People should avoid slaughtering and selling moribund animals. The carcass should be handled safely and appropriately buried to break the anthrax transmission cycle. The collaboration between animal and human health departments must be strengthened through effective coordination and cooperation at every level (national, regional and local) for early detection and responding to the anthrax outbreak using one‐health approach.

### 3.4 Rabies

Rabies is a highly fatal but vaccine‐preventable zoonotic disease caused by rabies virus of the genus Lyssavirus under *Rhabdoviridae* family (Fooks et al., 2014). The case fatality rate is 100% in untreated cases (Fooks et al., 2017). Rabies is prevalent worldwide. Globally rabies is maintained by dogs, foxes, raccoon dogs, skunks, jackals and bats (Bernardi et al., 2005; Fooks et al., 2017; Wunner & Jackson, 2010). Rabies can be prevented by vaccination in humans and animals. World Health Organization (WHO) approved three vaccines for humans (Anthony R. Fooks et al., 2017). WHO recommended at least 70% dog vaccination to control rabies (Davlin & VonVille, 2012). Rabies is endemic in the dog population in Bangladesh. Domestic stray dogs are the main reservoir for rabies. Bangladesh reported an estimated 2000–2500 annual death due to rabies in humans (Gongal & Wright, 2011). The majority (86%) of the human cases was associated with dog bites (Sumon Ghosh et al., 2016). More than 300,000 dog bites were recorded annually (Gongal & Wright, 2011). The government implemented a mass dog vaccination programme to reduce rabies infection in dogs and humans (Ghosh et al., 2020). The stray dog population increases every day (Tenzin et al., 2015). As most human cases were associated with dog bites, the government should focus on management of dog population and mass dog vaccination. Surgical sterilisation can be used to maintain the dog population. Currently, no rabies surveillance is going on. An integrated one‐health surveillance is useful to understand the current situation of rabies in animals (domestic and wildlife) and humans. More research is needed to identify the risk factors for rabies fatality among dog bite patients and to evaluate the effectiveness of the current vaccine in dogs and humans. Community awareness about rabies transmission, dog bite management, post‐exposure prophylaxis and dog vaccination is crucial to control rabies in humans and animals.

### 3.5 Japanese encephalitis

Japanese encephalitis (JE) is a zoonotic disease caused by the mosquito‐borne Flavivirus. JE is endemic in humans in South Asian countries, including Bangladesh, India, Pakistan, and Myanmar (Erlanger et al., 2009). Culex mosquito species transmit viruses from reservoir hosts to susceptible hosts such as pigs and humans (Solomon et al., 2000). It causes reproductive disorders in pigs. The case fatality rate in piglets may reach up to 100%. Swine act as an intermediate and amplifying host for JE. Wading ardeid water birds such as herons and egrets carry the virus as reservoir hosts (Solomon et al., 2000). Bangladesh reported the first JE case in humans in 1977 (Khan et al., 1981). Hospital‐based encephalitis surveillance detected that 4%‐6% of encephalitis patients had antibodies against JE (Hossain et al., 2010). Highest number of human cases was reported from Rajshahi region, the Northwest part of Bangladesh (Paul et al., 2011). An animal study found evidence of previous exposure to JE virus in domestic pigs (30%) in the same region (Khan et al., 2014). Domestic pigs could act as a reservoir of JE virus and contribute to transmission to humans by mosquitos. Till to date, Bangladesh conducted minimal studies to understand the detailed epidemiology of this disease. No published data are available so far to detect JE virus in reservoir hosts and mosquito populations. More research studies are necessary to understand the epidemiology of JE, the effectiveness of the JE vaccine in pigs and humans, the role of local pigs, the status of JE in local reservoir host species and molecular characterisation. Hospital and community‐based surveillance at high risk areas can be useful to monitor JE situation over the period.

### 3.6 Leptospirosis

Leptospirosis is a re‐emerging zoonotic disease caused by pathogenic spirochetes of the genus *Leptospira* (Bharti et al., 2003). Many species of rodents act as a reservoir of Leptospira. Among rodents, rats are mostly carrying and excrete leptospires. Reservoir hosts shed Leptospira in their urine. Domestic farm animals, dogs and humans can be exposed to Leptospira through contaminated water, food and soil. Leptospira causes acute fever, jaundice, acute renal failure and bleeding in humans (McBride et al., 2005). In animals, Leptospira mainly causes abortion, stillbirth and low milk production (Petrakovsky et al., 2014). Leptospira was first reported in Bangladesh in 1994 (Morshed et al., 1994). Several studies detected Leptospira species infection and antibodies in humans in Bangladesh (Aziz et al., 2019; Faruque et al., 2017; Kendall et al., 2010; LaRocque et al., 2005; Morshed et al., 1994). Antibodies were also detected in cattle (Parvez et al., 2015). A study found Leptospira in 13% of rodents in Bangladesh (Krijger et al., 2019). Leptospira remains neglected in Bangladesh because of the poor diagnostic facilities and limited understanding of the disease burden. Evidence of Leptospira in rodents indicates Leptospira infection is not uncommon. Bangladesh has all suitable conditions for Leptospira transmission, such as long monsoon, frequent flooding, stagnant water, high temperature, high humidity and regular animal–human contact. Laboratory testing capacity should be established and enhanced at all regions for rapid detection and treatment of Leptospira infection. More research is needed to understand the burden of Leptospira in humans and animals, to explore the role of rodents for Leptospira transmission and to assess the effectiveness of antibiotics used for treatment.

### 3.7 Pandemic H1N1 2009 (Swine flu)

A swine‐origin novel subtype of influenza virus A (H1N1) was first detected in humans in March 2009. Mexico reported the first outbreak of this virus (WHO, 2009). This novel virus is thought to be the result of reassortment of influenza A strain H1N1 from avian, swine and human strains (Coker, 2009). Pigs could serve as “mixing vessels” for this reassortment (Haß et al., 2011). More than 170 countries were affected by this virus in 2009 (Sebastian et al., 2009). Bangladesh detected the first human case in 2009 (Azziz‐Baumgartner et al., 2012). A study reported an estimated mean annual influenza‐associated mortality was 11 per 100,000 in Bangladesh (Homaira et al., 2012). So far, no studies in Bangladesh reported this novel virus in swine, poultry, and other animals. Countrywide surveillance is necessary to detect novel influenza strains of public health interest in the human–pig–poultry interface using one‐health approach.

### 3.8 Brucellosis

Brucellosis is a neglected zoonotic disease caused by the bacteria Brucella. *Brucella melitensis*, *Brucella abortus*, *Brucella suis* and *Brucella canis* cause illnesses in humans (Young, 1995). Most human cases were either because of occupationally exposed or consumption of unpasteurised dairy products. In humans, Brucellosis causes a chronic debilitating illness with fever, sweating, fatigue, weight loss, headache and joint pain (Dean et al., 2012). In livestock, abortion in the last trimester of gestation and infertility was commonly found (Olsen & Tatum, 2010). In Bangladesh, antibodies against *Brucella species* were detected in 3%–7% of livestock (Islam, Khatun, et al., 2013). Antibodies and Brucella isolates were detected in humans (Rahman et al., 1988, 2006, 2012). No studies on the effectiveness of Brucella vaccines and other interventions to control Brucellosis in livestock were published. More studies are needed to understand the epidemiology and burden of Brucellosis in humans and animals. Livestock workers, artificial inseminators, abattoir workers and animal practitioners are at high risk of getting Brucella infection. Appropriate use of personal protection equipment by animal health workers and practitioners during insemination and parturition of animals should be ensured to avoid Brucella exposure.

### 3.9 Zoonotic Rotavirus

Rotavirus‐associated enteric infection is the common cause of diarrhea in children and livestock (Martella et al., 2010). Rotavirus infection is mostly host‐specific, but the human can be infected by animal rotaviruses (Cook et al., 2004). Several studies identified host‐specific rotavirus infection in humans and animals in Bangladesh (Hossain et al., 2020; Satter et al., 2017). Zoonotic transmission of rotavirus was not frequently reported in Bangladesh. Very few studies detected animal‐like rotaviruses such as bovine‐like human VP4 mono‐reassortant G6P(8) and novel G11P(25) rotavirus in humans (Afrad et al., 2013). Though rotavirus transmission from animal to human is infrequent, the animal origin reassortant rotavirus can emerge in humans. Rotavirus surveillance is necessary to detect novel reassortant strains of rotaviruses.

### 3.10 Zoonotic tuberculosis

Zoonotic tuberculosis in humans is caused mostly by *Mycobacterium bovis*. Cattle are the main reservoir for zoonotic tuberculosis. Transmission of *Mycobacterium bovis* in humans mostly occurred through close contact with infected cattle and the consumption of unpasteurised milk (Müller et al., 2013). Zoonotic tuberculosis in humans is characterised by cervical lymphadenopathy, intestinal lesions and chronic skin lesion (lupusvulgaris; Cosivi et al., 1998). Few studies reported *Mycobacterium bovis* in cattle in Bangladesh (Rahman & Samad, 2008; Sarker et al., 2015). So far, only one study detected *Mycobacterium bovis* in humans in Bangladesh (Rahman et al., 2015). Zoonotic tuberculosis remains neglected in Bangladesh. Evidence of *Mycobacterium bovis* in human samples indicates the risk of zoonotic transmission. Routine testing of M*ycobacterium bovis* in the human population can provide a better understanding of the risk of zoonotic tuberculosis transmission.

### 3.11 Toxoplasmosis

Toxoplasmosis is a water‐borne zoonotic disease caused by an obligate intracellular protozoan parasite *Toxoplasma gondii* (Halonen & Weiss, 2013). Humans become infected with this protozoa by eating contaminated food or water with oocysts from the faeces of infected cats (Dubey, 2004). *Toxoplasma gondii* cause fever, lymphadenopathy and seizures. Infected pregnant women can transmit the infection to the fetus through the placenta, and fetal death and premature birth may happen. Toxoplasmosis is mostly asymptomatic in the cat. In sheep, goats and pigs, it causes mainly stillbirth (Aguirre et al., 2019). Few studies reported seroprevalence of *Toxoplasma gondii* in human (15%–39%) and animals (8%–70%) in Bangladesh (Naheen et al., 2018; Rahman, Rahman, et al., 2018; Sah et al., 2019; Samad et al., 1993). A study detected *Toxoplasma gondii* in 10% of rodents (Krijger et al., 2019). So far, no published data are available on the epidemiology of Toxoplasmosis in the cat. Toxoplasmosis remains neglected in Bangladesh because of limited diagnostic facilities and low interest. More research studies are needed to generate more information on Toxoplasmosis in humans and animals.

### 3.12 COVID‐19

The coronavirus disease 19 (COVID‐19) is a highly transmissible disease infecting more than four million people worldwide as of 12 May 2020 (WHO, 2020b). COVID‐19 is caused by severe acute respiratory syndrome coronavirus 2 (SARS‐CoV‐2). World Health Organization (WHO) reported the first outbreak on 31 December 2019. The virus was first detected in the city of Wuhan in Hubei Province, China, and the first outbreak was epidemiologically linked with wet animal and sea food market (Mackenzie & Smith, 2020). SARS‐CoV‐2 is thought to be originated from wild animals, possibly a bat coronavirus (Zhou et al., 2020). The virus has 96.2% nucleotide homology with a bat‐origin CoV RaTG13 virus (Ye et al., 2020). As of 13 May 2020, Bangladesh reported 17,822 cases since 8 March 2020 (DGHS, 2020). From 21 January 2020 to 13 May 2020, a total of 144,538 samples were tested to detect SAR‐CoV‐2 using RT‐PCR. The case fatality rate is 3.5% (DGHS, 2020). Active epidemiological surveillance is needed for rapid case detection and contains spread in humans. More research is needed to characterise the local SARS‐CoV‐2 virus and examine genotypic evolution. Wildlife and live animal markets require active monitoring to detect emerging pathogens including novel coronaviruses of public health importance.

### 3.13 Tick and flea‐borne zoonoses

Tick‐borne zoonotic are mainly caused by *Coxiella burnetii*, *Rickettsia rickettsii*, *Borrelia burgdorferi*, *Francisella tularensis*, tick‐borne encephalitis virus, Kyasanur forest disease (KFD) virus and Crimean–Congo haemorrhagic fever (CCHF) virus (Sambri et al., 2004). Tick‐borne encephalitis and Kyasanur forest disease viruses are the members of the genus Flavivirus of the family *Flaviviridae*. Crimean–Congo haemorrhagic fever virus is a member of the genus Nairovirus of the family *Bunyaviridae*. KFD and CCHF have caused multiple outbreaks in humans in India (Mourya et al., 2014). Wild and domestic animals act as a reservoir for tick‐borne zoonotic pathogens. *Ixodid* spp, *Dermacentor* spp, *Hyalomma anatolicum anatolicum*, *Haemaphysalis spinigera* are mostly transmitting zoonotic pathogens (Mourya et al., 2014). Bangladesh reported flea‐borne *Rickettsia felis* infection in humans and tick‐borne *Coxiella burnetii* infection in cattle and goats (Chakrabartty et al., 2016; Ferdouse et al., 2015; Haider et al., 2015; Rahman, Chakrabartty, et al., 2018). Although Bangladesh has many potential ticks, including *Ixodid* spp, *Haemaphysalis* spp, *Hyalomma* spp, no human cases were reported yet for tick‐borne encephalitis virus, KFD and CCHF (Ghosh et al., 2007). However, the geographical location and abundance of tick population make peoples always at risk for tick‐borne encephalitis virus, KFD and CCHF. Bangladesh has conducted very minimal studies to detect tick and flea‐borne diseases of public health interest. Nationwide surveillance may be useful to detect potential tick and flea‐borne pathogens in animals and humans.

### 3.14 Food‐borne zoonoses

Verotoxigenic *E. coli* (VTEC), *Salmonella* spp, *Campylobacter* spp and *Listeria monocytogenes* are considered as the major causes of food‐borne illness. The majority of the pathogens causing food‐borne illness are zoonotic (Schlundt et al., 2004). Food‐borne diseases are likely one of the most severe public health problems in Bangladesh. Hospital‐based surveillance reported that enterotoxigenic *E. coli* (20%), rotavirus (19%), *Campylobacter jejuni* (14%), Vibrio cholera (6%), Shigella (12%) and Salmonella (1%) were the main causes of diarrhoea in Bangladesh (Stoll et al., 1982). Multiple studies detected food‐borne pathogens such as Salmonella, Campylobacter, *E. Coli* O157, Shigella, *Listeria monocytogenes* and *Echinococcus granulosus* in domestic animals in Bangladesh (Barua et al., 2014; Faruk et al., 2017; Islam, 1982; Islam et al., 2016). Salmonella, Campylobacter, *E. Coli* O157, Shigella, and *Echinococcus granulosus* were detected in humans (Karim et al., 2015). Studies detected Shiga toxin‐producing *Escherichia coli* and Salmonella in raw meat and milk (Al‐Salauddin et al., 2015; Mohammad A Islam et al., 2010). Moreover, antibiotics are heavily used in animals that may lead to accelerating antibiotic resistance (Amin et al., 2020; Hasan et al., 2011; Kabir et al., 2018). Multidrug‐resistant animal origin pathogens may be transmitted to humans through the consumption of contaminated foods. An integrated one‐health approach surveillance is necessary to monitor antimicrobial‐resistant pathogens in humans and animals.

### 3.15 Other potential zoonotic diseases

There are some other potential zoonotic viruses such as Middle East respiratory syndrome coronavirus (MERS‐CoV), Ebola, West Nile virus (WNV), zoonotic hepatitis E virus, and zoonotic pox virus, which can be emerged to human in Bangladesh. People of Bangladesh live very close to domestic and peri‐domestic animals such as rodents, crows and bats. A study detected antibodies for MERS‐CoV in camel raised in Bangladesh (Islam et al., 2018). Antibodies against Ebola Zaire and Reston viruses were detected in bats in Bangladesh (Olival et al., 2013). Though Bangladesh has not reported any mosquito‐borne WNV, India detected antibodies against WNV in humans and isolated WNV in mosquitoes (Khan et al., 2011; Paramasivan et al., 2003). Zoonotic poxviruses (Parapoxvirus) were reported for the first time in Bangladesh in cows (Lederman et al., 2014). So far, very limited studies were conducted to detect these pathogens in Bangladesh. Further studies are needed to assess the risk of transmission of these pathogens to humans. Countrywide wildlife surveillance can be very useful to detect zoonotic pathogens of public health interest.

## 4 CONCLUSIONS

Bangladesh has generated adequate information on the epidemiology of certain zoonotic diseases such as the nipah and highly pathogenic avian influenza. Information about other potential zoonotic diseases is limited. Only a few studies were conducted to develop and design locally accepted and effective interventions to control zoonotic disease transmission. Very few molecular and immunologic studies were performed so far. More research studies are needed to understand the burden of zoonotic diseases and to develop interventions. People of Bangladesh are not well conscious about zoonotic diseases and associated risk factors. Awareness of zoonotic diseases, proper biosafety measures, improved biosecurity practices, the impact of deforestation and wildlife hunting, wildlife conservation, safe animal handling and carcass disposal must be improved in general people through mass communication. Strong multi‐sectoral collaboration among medical professionals, veterinarians, eco‐health and agriculture personals is required to detect, respond, and prevent zoonotic diseases in Bangladesh using one‐health approach.

## CONFLICT OF INTEREST

The authors have no conflict of interest to declare.

## AUTHOR CONTRIBUTION

**Sukanta Chowdhury:** Conceptualization; Data curation; Formal analysis; Methodology; Project administration; Resources; Software; Validation; Writing‐original draft. **Mohammad Abdul Aleem:** Conceptualization; Data curation; Formal analysis; Methodology; Writing‐review & editing. **Md Shafiqul Islam Khan:** Conceptualization; Formal analysis; Methodology; Writing‐review & editing. **Mohammad Enayet Hossain:** Conceptualization; Methodology; Writing‐review & editing. **Sumon Ghosh:** Conceptualization; Formal analysis; Writing‐review & editing. **Mohammed Ziaur Rahman:** Conceptualization; Formal analysis; Methodology; Supervision; Writing‐review & editing.

### PEER REVIEW

The peer review history for this article is available at https://publons.com/publon/10.1002/vms3.465.

## Data Availability

The data of this study are available from the corresponding author upon reasonable request.
